# Glucocorticoids limit acute lung inflammation in concert with inflammatory stimuli by induction of SphK1

**DOI:** 10.1038/ncomms8796

**Published:** 2015-07-17

**Authors:** Sabine Vettorazzi, Constantin Bode, Lien Dejager, Lucien Frappart, Ekaterina Shelest, Carina Klaßen, Alpaslan Tasdogan, Holger M. Reichardt, Claude Libert, Marion Schneider, Falk Weih, N. Henriette Uhlenhaut, Jean-Pierre David, Markus Gräler, Anna Kleiman, Jan P. Tuckermann

**Affiliations:** 1 Institute of Comparative Molecular Endocrinology (CME), Ulm University, 89081 Ulm, Germany; 2 Leibniz Institute for Age Research – Fritz Lipmann Institute, 07745 Jena, Germany; 3 Molecular Cancer Research Centre (MKFZ), Charité – University Medical School (CVK), 13353 Berlin, Germany; 4 Inflammation Research Center, Mouse Genetics in Inflammation group, VIB and University Ghent, B9052 Ghent, Belgium; 5 Department of Pathology, Bat 10, HCL-Edouard Herriot Hospital, INSERM U590, 69437 Lyon, France; 6 Leibniz Institute for Natural Product Research and Infection Biology Hans Knöll Institute (HKI), 07745 Jena, Germany; 7 Institute for Cellular and Molecular Immunology, University of Göttingen Medical School, 37073 Göttingen, Germany; 8 Institute for Immunology, Ulm University, 89081 Ulm, Germany; 9 Section of Experimental Anesthesiology, University Clinic Ulm, 89081 Ulm, Germany; 10 Institute for Diabetes and Obesity, Helmholtz Zentrum München, 85748 Garching, Germany; 11 Department of Osteology and Biomechanics, University Medical Center Hamburg-Eppendorf, 20246 Hamburg, Germany; 12 Department of Anesthesiology and Intensive Care Medicine, Center for Sepsis Control and Care (CSCC) and Center for Molecular Biomedicine (CMB), University Hospital Jena, 07740 Jena, Germany

## Abstract

Acute lung injury (ALI) is a severe inflammatory disease for which no specific treatment exists. As glucocorticoids have potent immunosuppressive effects, their application in ALI is currently being tested in clinical trials. However, the benefits of this type of regimen remain unclear. Here we identify a mechanism of glucocorticoid action that challenges the long-standing dogma of cytokine repression by the glucocorticoid receptor. Contrarily, synergistic gene induction of sphingosine kinase 1 (*SphK1*) by glucocorticoids and pro-inflammatory stimuli via the glucocorticoid receptor in macrophages increases circulating sphingosine 1-phosphate levels, which proves essential for the inhibition of inflammation. Chemical or genetic inhibition of *SphK1* abrogates the therapeutic effects of glucocorticoids. Inflammatory p38 MAPK- and mitogen- and stress-activated protein kinase 1 (MSK1)-dependent pathways cooperate with glucocorticoids to upregulate *SphK1* expression. Our findings support a critical role for *SphK1* induction in the suppression of lung inflammation by glucocorticoids, and therefore provide rationales for effective anti-inflammatory therapies.

## Abstract (web summary)

Endothelial damage is a major component of acute lung injury pathogenesis. Here the authors show that in a mouse model of acute lung injury, glucocorticoids induce sphingosine kinase 1 production in macrophages, promoting endothelial barrier function and ameliorating the disease.

## Graphical abstract

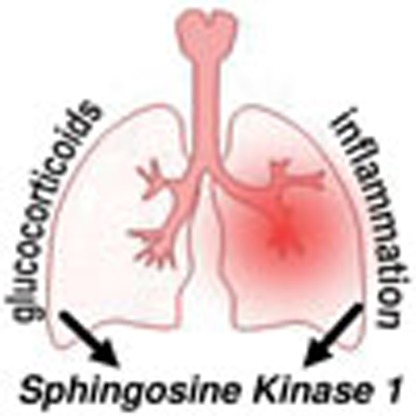

Acute lung injury (ALI) and acute respiratory distress syndrome (ARDS) are severe inflammatory lung diseases induced by infectious insults. Both syndromes may also develop as complications of severe trauma or sepsis^1^. Forty per cent of septic patients develop ALI, of whom about half die from inflammatory lung diseases^2^,^3^. Despite the potent anti-inflammatory effects of glucocorticoids (GCs), their use to treat or prevent ALI and ARDS is controversially discussed in the clinic^4^,^5^. GC treatment in the early phase of lung inflammation appears to resolve ALI and ARDS^6^,^7^,^8^. A larger clinical study is currently trying to determine the parameters for more effective GC treatment and to develop appropriate guidelines (clinical trial NCT01731795). One major obstacle facing the use of GCs is the inadequate understanding of the molecular mechanisms by which they resolve lung inflammation. Therefore, a precise definition of the cell type-specific mode of GC action in the context of ALI is essential for rational drug design and treatment regimens that lead to effective resolution of inflammation in ALI and ARDS.

GCs act by binding their cognate receptor, the GC receptor (GR), a member of the nuclear hormone receptor family. As a monomer, ligand-activated GR represses the activities of pro-inflammatory transcription factors, such as activator protein 1, nuclear factor kappa B (NF-κB) and interferon regulatory factor 3, by a tethering mechanism called transrepression^9^,^10^. On the other hand, the direct binding of dimerized GR to response elements in the DNA (GC-response elements) induces gene transcription, commonly referred to as transactivation. The prevailing view is that transrepression by the GR monomer is a major mechanism for inhibiting inflammation^11^. However, it was recently shown that anti-inflammatory responses require not only gene repression but also gene activation. Indeed, mice with a point mutation in the GR DNA-binding domain (*GR^dim^*) exhibit less transactivation of GC-induced genes *in vivo*^12^ and fail to resolve inflammation in allergic^13^, autoimmune^14^ and systemic inflammation^15^,^16^. This is partly because of the inability of GCs to induce anti-inflammatory genes, such as dual specificity phosphatase 1 (*Dusp1*; refs 15, 17) and annexin A1 (*Anxa1*; ref. 18). However, the mechanism and the target cells that mediate the anti-inflammatory effects of GCs in lung inflammation are unknown.

A hallmark of ALI is disruption of the endothelial barrier that facilitates massive infiltration of leukocytes into the alveolar space^19^. A major regulatory system of endothelial barrier integrity in the lung is sphingosine kinase 1 (*SphK1*) and its enzymatic product, sphingosine 1-phosphate (S1P). The active mediator S1P is synthesized from sphingosine by *SphK1* (ref. 20) and binds to a family of receptors, of which S1P receptor type 1 (S1PR1) is of particular importance because it is expressed on endothelial cells^21^. S1PR1 triggers Rho family-dependent cytoskeletal reorganization, leading to enhancement of the barrier function of the endothelium^21^. This in turn prevents vascular leakage and massive infiltration in mouse models of ALI^22^,^23^,^24^,^25^.

Here we demonstrate that GCs attenuate inflammation in a murine model of ALI by synergistic upregulation of *SphK1* gene expression in macrophages. This is followed by elevation of plasma S1P levels, thereby leading to protection of the endothelial barrier function and to amelioration of the disease.

## Results

### GCs suppress ALI via the GR dimerization in myeloid cells

**Figure 1:**
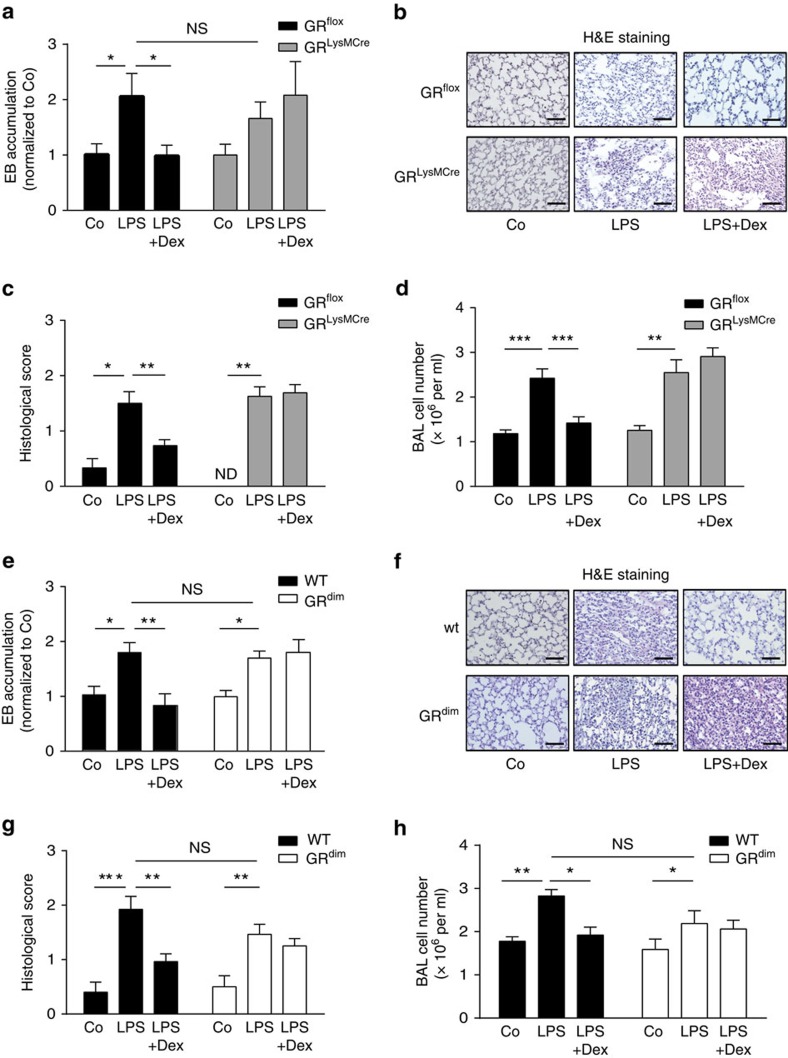
GCs inhibit lung inflammation by the GR in myeloid cells requiring an intact GR dimerization interface. (**a**) *GR*^*flox*^ and *GR*^*LysMCre*^ mice were injected with vehicle (Co), LPS and OA (hereafter, the LPS and OA co-treatment will be designated as ‘LPS') or LPS and Dex (LPS+Dex). After 18 h, EB was injected i.v., and the mice were killed 30 min later. EB accumulation in the lung was determined after perfusion with PBS+5 mM EDTA. (**b**) Representative haematoxylin/eosin (H&E) staining of lung sections (scale bars, 0.1 mm), and (**c**) histological scores of *GR*^*flox*^ and *GR*^*LysMCre*^ mice treated as described in **a**. (**d**) *GR*^*flox*^ and *GR*^*LysMCre*^ mice were treated as described in **a**. Cell numbers were determined in the BAL 18 h after the indicated treatments. (**e**) *GR*^*dim*^ and wild-type (WT) littermate mice were injected with vehicle (Co), LPS or LPS+Dex. After 24 h, EB was injected i.v. and 30 min later the mice were killed. EB accumulation in the lung was quantified as described in **a**. (**f**) Representative H&E staining of lung sections (scale bars, 0.1 mm) and (**g**) histological scores of lungs of littermate WT and *GR*^*dim*^ mice from the experiment described in **e**. (**h**) *GR*^*dim*^ and WT mice were treated as described in **e**. Cell numbers were determined in the BAL 24 h after the indicated treatments. Results are presented as mean±s.e.m. Number of biological replicates: (**a**) (6–10), (**c**) (18–20), (**d**) (7–13), (**e**) (4–8), (**g**) (12–16) and (**h**) (7–11). In **a**, data are from three independent biological experiments; in **c**, data are from four independent biological experiments; in **d**, data are from two independent biological experiments; in **e**, data are from two independent biological experiments; in **g**, data are from three independent biological experiments out of four; and in **h**, data are from two independent biological experiments. Statistical analysis was performed by one-way analysis of variance. *P<0.05, **P<0.01, ***P<0.001; ND, not detectable; NS, not significant.

ALI was induced by systemic lipopolysaccharide (LPS) administration followed by application of oleic acid (OA; hereafter, co-treatment with both compounds is designated ‘LPS'). OA was used as an additional trigger for lung inflammation because naturally occurring serum OA has been shown to be elevated in samples from sepsis patients at risk of developing lung inflammation^26^,^27^. The GR agonist dexamethasone (Dex) was applied during ALI to investigate the anti-inflammatory actions of GCs. LPS treatment resulted in strong lung inflammation (Fig. 1) that was characterized by two features: (i) a strong vascular leakage, as determined by the accumulation of Evans Blue dye (EB) in lung tissue (Fig. 1a) and (ii) an enhanced inflammatory profile, as characterized by increased leukocyte infiltration into the lung (Fig. 1b–d). These two features are also hallmarks of human ALI^28^,^29^,^30^. It has previously been reported that myeloid cells play an important role in mediating the anti-inflammatory effects of GCs during sepsis and are critical for survival during inflammation^16^,^17^,^31^. For this reason, myeloid cells might also be involved in the suppression of ALI by GCs. To test this hypothesis, *Nr3c1*^*tm2Gsc*^*Lyz2tm1(cre)lfo/J* (hereafter referred to as *GR*^*LysMCre*^) mice lacking the GR in myeloid cells were subjected to ALI in the presence or absence of Dex (Fig. 1a–d). Endothelial barrier permeability in unchallenged mice, as measured by EB accumulation, was the same in mutants (*GR*^*LysMCre*^) and *Nr3c1*^*tm2Gsc*^ controls (hereafter referred to as *GR*^*flox*^; Supplementary Fig. 1a), and it was similarly affected by LPS treatment in both strains (Fig. 1a). Concomitantly, *GR*^*LysMCre*^ and control wild-type (*GR*^*flox*^) mice responded to LPS with a strong cellular infiltration and inflammation in the lung (Fig. 1b–d). Treatment of LPS-challenged *GR*^*flox*^ mice with Dex (LPS+Dex) reduced EB accumulation (Fig. 1a) and decreased leukocyte infiltration (Fig. 1b–d). In contrast to control mice, LPS-challenged *GR*^*LysMCre*^ mice were refractory to Dex treatment. EB accumulation was not reduced (Fig. 1a), cellular infiltration was persistent (Fig. 1b,c) and bronchoalveolar lavage (BAL) cell numbers were unaltered (Fig. 1d). Accordingly, the histological score reflecting the severity of ALI^32^ was reduced after Dex treatment in *GR*^*flox*^ control mice but not in *GR*^*LysMCre*^ mice (Fig. 1c). Thus, the GR in myeloid cells is required for the suppression of ALI pathology by GCs.

GR deficiency in *GR*^*LysMCre*^ mice affects both macrophages and neutrophils. To exclude the possibility that the GR in neutrophils makes a major contribution to lung endothelial stability, *GR*^*loxP*^ mice were crossed with *Nr3c1*^*tm2Gsc*^*Tg(Itgax-cre)1-1Reiz* (hereafter referred to as *CD11cCre*) mice^33^. The resultant *GR*^*CD11cCre*^ mice did not show any recombination of the GRloxP allele in neutrophils, but there was efficient recombination in blood monocytes (Supplementary Fig. 1b). Importantly, *GR*^*CD11cCre*^ mice were resistant to Dex treatment during ALI in comparison with wild-type littermates (*GR*^*flox*^) in terms of vascular leakage (Supplementary Fig. 1c), indicating that loss of the GR in monocytes (Supplementary Fig. 1b) is sufficient to confer resistance to Dex treatment in ALI (Supplementary Fig. 1c). Finally, we tested whether an intact GR dimerization interface is required for the anti-inflammatory activity of GCs during ALI. To this end, *Nr3c1*^*tm3Gsc*^ (hereafter referred to as *GR*^*dim*^) mice were subjected to ALI and treated with Dex. This treatment failed to reduce EB accumulation in the lung (Fig. 1e), cellular infiltration (Fig. 1f), the histological score (Fig. 1g) and cell numbers in the BAL fluid (Fig. 1h) in *GR*^*dim*^ mice. The extent of the LPS-induced lung injury assessed by endothelial permeability, histological score and cell numbers in the BAL fluid was similar in wild-type and mutant mice (no significant differences; Fig. 1e,g,h). This implies that a functional GR dimerization interface is required for the repression of inflammation in ALI by GCs.

Of note, absolute numbers (Supplementary Fig. 2) of major leukocyte subpopulations in the blood were similar in mice of both genotypes. The only exception was the abundance of neutrophil granulocytes, which was elevated by Dex application in wild-type mice, while it was diminished in *GR*^*dim*^ mice (Supplementary Fig. 2a).

### Dex elevates circulating S1P dependent on GR in macrophages

**Figure 2:**
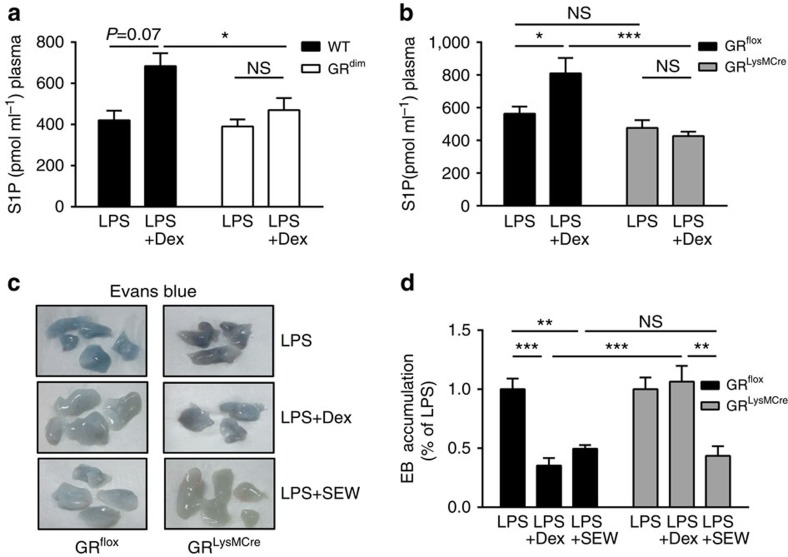
S1P signalling increased by Dex or S1PR1 agonist reduces ALI in GR^*LysMCre*^ mice. (**a**) *GR*^*dim*^ and wild-type (WT) littermate control mice were treated as described in Fig. 1e. The S1P level in plasma (pmol ml^−1^) was determined by LC/MS/MS after 24 h. (**b**) *GR*^*flox*^ and *GR*^*LysMCre*^ mice were treated as described in Fig. 1a. The S1P level in plasma (pmol ml^−1^) was determined by LC/MS/MS after 18 h. (**c**) *GR*^*flox*^ and *GR*^*LysMCre*^ mice were treated as described in Fig. 1a, and an additional group of LPS-treated mice received SEW2781 (LPS+SEW). EB was injected i.v. 30 min before killing. Representative lungs are shown to demonstrate EB accumulation. (**d**) Quantification of EB accumulation of the experiment described in **c** determined as described in Fig. 1a. Results are presented as mean±s.e.m. Number of biological replicates: (**a**) (9), (**b**) (15–20) and (**d**) (9 or 10). Data in **a** are from two independent biological experiments, and data from **b** and **d** are from three independent biological experiments. Statistical analysis was performed by one-way analysis of variance. *P<0.05, **P<0.01 and ***P<0.001; NS, not significant.

A primary mechanism of ALI pathogenesis is severe impairment of endothelial barrier integrity in the lung. A critical role for the sphingolipid S1P in maintaining lung endothelial barrier function has been well demonstrated^21^,^34^. Consequently, we hypothesized that plasma S1P levels might be influenced by Dex treatment in ALI. No change in circulating S1P levels was detectable in LPS-induced ALI; however, Dex treatment during ALI significantly increased circulating plasma S1P levels (Supplementary Fig. 1d). In contrast to their respective control mice in which plasma S1P levels were increased after treatment of ALI with Dex, mice with an impaired GR dimerization interface (*GR*^*dim*^; Fig. 2a and Supplementary Fig. 3a) and mice with deletion of the GR in myeloid cells (*GR*^*LysMCre*^; Fig. 2b and Supplementary Fig. 3b) displayed no change in plasma S1P levels. LPS-induced ALI did not cause a significant change in plasma S1P levels between *GR*^*LysMCre*^ mice and littermate control mice (Fig. 2b). We conclude that GCs elevate plasma S1P levels only if GR dimerization and the GR in macrophages are intact. Specific activation of S1PR1 with SEW2781 strengthened the endothelial barrier in *GR*^*LysMCre*^ mice. SEW2781 was as effective as Dex in reducing EB accumulation in LPS-challenged wild-type mice, and it rescued the failure of *GR*^*LysMCre*^ mice to respond to Dex's beneficial effect on endothelial barrier integrity in the lung (Fig. 2c,d).

### Macrophage SphK1 is crucial for anti-inflammatory GC actions

As mice lacking GR expression in macrophages failed to upregulate S1P levels, we tested the contribution of *SphK1* in these cells to S1P generation and its involvement in the observed anti-inflammatory effects of GCs. To this end, conditional myeloid cell-specific *SphK1* knockout mice (*SphK1*^*tm2Cgh*^*Lyz2tm1(cre)lfo/J*; hereafter referred to as *SphK1*^*LysMCre*^) were generated and analysed. In these mice, expression of SphK1 in isolated macrophages was diminished (Supplementary Fig. 4a). In contrast, circulating leukocytes were not altered in *SphK1*^*LysMCre*^ mice in comparison with *SphK1*^*flox*^ (*SphK1*^*tm2Cgh*^; hereafter referred to as *SphK1*^*flox*^) controls (Supplementary Fig. 4b,c).

**Figure 3:**
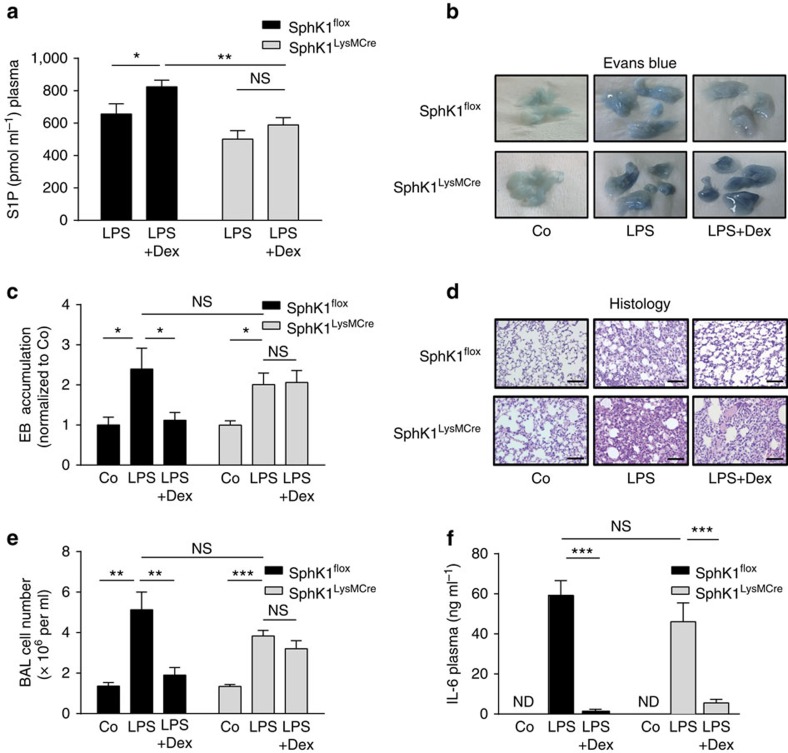
SphK1 expression in myeloid cells is essential for the anti-inflammatory effects of GCs in ALI. (**a**) *SphK1*^*flox*^ and *SphK1*^*LysMCre*^ mice were treated as described in Fig. 1a. The S1P level in plasma (pmol ml^−1^) was determined 24 h later by LC/MS/MS. (**b**) *SphK1*^*flox*^ and *SphK1*^*LysMCre*^ mice were treated as described in Fig. 1a, and lung specimens with accumulated EB are shown. (**c**) EB accumulation of isolated lungs was quantified as described in Fig. 1a. (**d**) Representative haematoxylin/eosin staining of lung sections from *SphK1*^*flox*^ and *SphK1*^*LysMCre*^ mice treated as described in Fig. 1a (scale bars, 0.1 mm). (**e**) Cell numbers were determined in the BAL 24 h after the indicated treatments. (**f**) IL-6 plasma levels (ng ml^−1^) in *SphK1*^*flox*^ and *SphK1*^*LysMCre*^ mice were determined by ELISA 5 h after LPS or LPS+Dex treatment. Results are presented as mean±s.e.m. Number of biological replicates: (**a**) (8–15), (**c**) (5–12), (**e**) (5–12) and (**f**) (5–12). In **a**, **c** and **e**, the data are from two independent biological experiments; and in **f**, the data are from one biological experiment. Statistical analysis was performed by one-way analysis of variance. *P<0.05, **P<0.01 and ***P<0.001; ND, not detectable; NS, not significant.

In *SphK1*^*LysMCre*^ mice subjected to ALI, Dex did not increase circulating S1P levels (Fig. 3a and Supplementary Fig. 3c). This indicates that myeloid-specific SphK1 expression is responsible for the GC-induced increase in S1P levels during ALI. In accordance, Dex treatment of *SphK1*^*LysMCre*^ mice during ALI did not prevent endothelial leakage, as indicated by EB accumulation (Fig. 3b,c). Consequently, *SphK1*^*LysMCre*^ mice suffered from persistent inflammation and cellular infiltration during ALI even in the presence of Dex (Fig. 3d,e). The extent of the LPS-induced lung injury assessed by endothelial permeability and cell numbers in the BAL fluid was similar in wild-type and mutant mice (no significant differences; Fig. 3c,e).

By using the pharmacological SphK1 inhibitor *N*,*N*-dimethylsphingosine (DMS), we confirmed that *SphK1* is required for the substantial attenuation of lung inflammation by Dex. DMS fully abrogated the Dex-specific protective effects on endothelial barrier integrity and its suppression of cellular infiltration (Supplementary Fig. 4d,e).

Interestingly, we found that the inability of Dex to trigger anti-inflammatory actions in *SphK1*^*LysMCr*e^ mice was unrelated to cytokine secretion. Circulating interleukin (IL)-6 plasma levels were reduced in *SphK1*^*LysMCre*^ mice as efficiently as in wild-type mice and LPS-induced injury resulted in a similar IL-6 plasma induction in both genotypes (Fig. 3f). In line with this observation, IL-1β and iNOS expression were similarly reduced by Dex treatment in LPS-stimulated wild-type bone marrow-derived macrophages (BMDMs) and SphK1-deficient BMDMs (Supplementary Fig. 4f,g).

In conclusion, SphK1 expression in macrophages is a major mediator of GC effects on the integrity of the endothelial barrier and cellular infiltration, but is dispensable for Dex-mediated regulation of cytokines.

### Dex and pro-inflammatory stimuli synergistically induce SphK1

**Figure 4:**
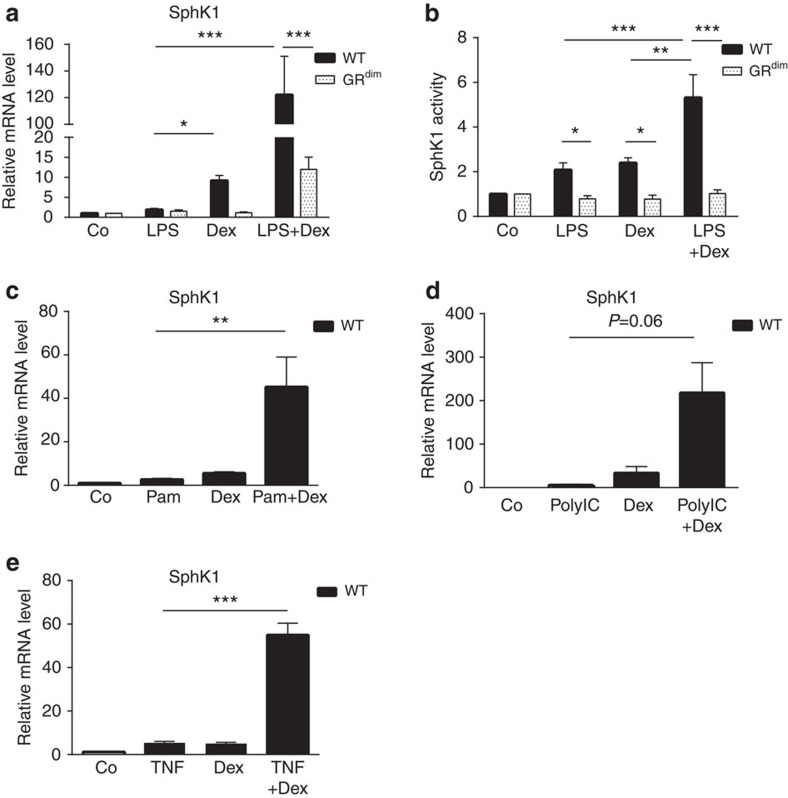
Dex and inflammatory stimuli synergistically elevate SphK1 mRNA and activity dependent on the GR in macrophages. (**a**) BMDMs derived from wild-type (WT) and *GR*^*dim*^ mice were treated with vehicle (Co), LPS, Dex or LPS+Dex for 4 h. SphK1 mRNA expression was analysed by quantitative RT–PCR. (**b**) BMDMs derived from WT and *GR*^*dim*^ mice were treated as described in **a** for 4 h. SphK1 activity was determined by the conversion of C17- and C18-sphingosine to S1P by LC/MS/MS. (**c**–**e**) BMDMs derived from WT mice were treated for 2 h with vehicle (Co), (**c**) Pam3CSK4 (TLR1/2 agonist), (**d**) polyIC (TLR3 agonist), (**e**) TNFα, Dex, or co-treatment with (**c**) Pam3CSK4 and Dex (Pam+Dex), (**d**) polyIC and Dex (polyIC+Dex), (**e**) TNFα and Dex (TNF+Dex). SphK1 mRNA expression was analysed by quantitative RT–PCR. Results are presented as mean±s.e.m. Number of biological replicates: (**a**) (5 or 6), (**b**) (4–8), (**c**) (5–7), (**d**) (3–5) and (**e**) (6 or 7). Statistical analysis was performed by one-way analysis of variance. *P<0.05, **P<0.01 and ***P<0.001.

Having shown that the GR and SphK1 in macrophages are essential for GC-mediated suppression of ALI, we next investigated whether an intact GR dimerization interface regulates SphK1 expression in primary macrophages. LPS treatment alone induced a twofold increase in SphK1 mRNA irrespective of the genotype. However, Dex exposure led to rapid upregulation of SphK1 mRNA in wild-type BMDMs but not in *GR^dim^* BMDMs (Fig. 4a). Intriguingly, combined treatment with LPS and Dex (LPS+Dex) elevated SphK1 mRNA expression even further in wild-type BMDMs (Fig. 4a). The synergistic increase of SphK1 mRNA levels after this combined treatment was evident after 1, 4 and 8 h (Fig. 4a and Supplementary Fig. 5a) and with various LPS/Dex concentrations (Supplementary Fig. 5b). Moreover, the observed effect was strongly attenuated in *GR^dim^* cells as well as in fetal liver cells derived from GR knockout macrophages (*GR^null^*), which entirely lack the GR (Fig. 4a and Supplementary Fig. 5a–c). Synergistic regulation of SphK1 expression was not observed in endothelial cells, another potential source of S1P. In both the TC10 cell line and primary lung endothelial cells, the Dex-induced SphK1 mRNA expression was not further stimulated by LPS (Supplementary Fig. 5d,e).

We then tested whether synergistic induction of the *SphK1* gene also extends to increased enzymatic activity of SphK1. In wild-type but not in *GR^dim^* BMDMs, enzymatic SphK1 activity was induced significantly more strongly in the combined presence of LPS and Dex than after separate treatment (Fig. 4b).

Finally, we asked whether the synergistic induction of SphK1 mRNA is restricted to augmenting Dex with LPS, or whether other strong pro-inflammatory triggers might also upregulate SphK1 expression in concert with Dex. To this end, we treated BMDMs with Dex and either the toll-like receptor 2 (TLR2) agonist Pam3CSK4 (Pam; Fig. 4c), the TLR3 agonist polyinosinic-polycytidylic acid (polyIC; Fig. 4d), or the cytokine tumour-necrosis factor-α (TNFα; Fig. 4e). In all cases, treatment with these pro-inflammatory triggers combined with Dex led to a synergistic elevation of SphK1 mRNA expression. Thus, effective activation of the anti-inflammatory *SphK1* gene by GCs can be achieved in concert with TLR2, -3 and -4 activators, as well as with TNFα, all of which trigger pro-inflammatory signalling pathways.

### Synergistic SphK1 induction is dependent on p38 MAPK activity

**Figure 5:**
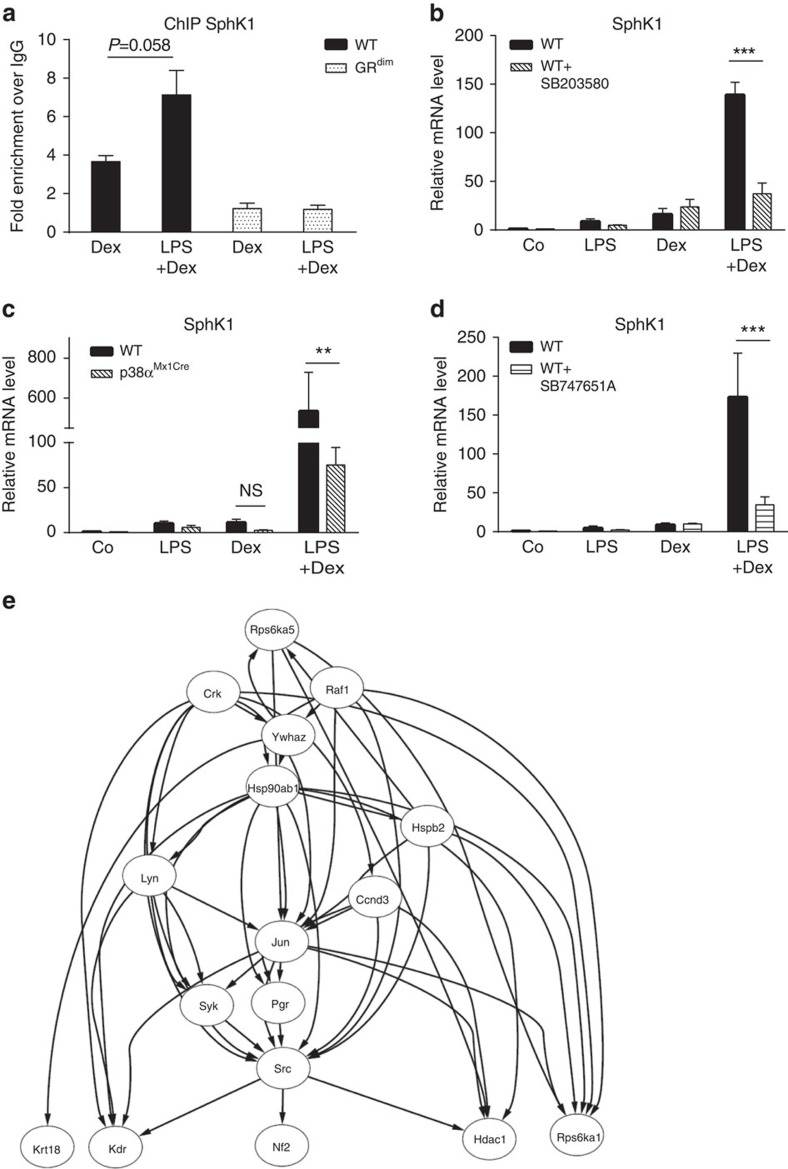
Dex and LPS treatment synergistically elevate SphK1 mRNA dependent on the p38 MAPK–MSK1 pathway. (**a**) ChIP of wild-type (WT) and *GR*^*dim*^ BMDMs was conducted 4 h after the indicated treatment (Dex, LPS+Dex). Shown are the quantitative RT–PCR results with SphK1 primer. (**b**) Quantitative analysis of SphK1 mRNA expression in BMDMs treated for 2 h with vehicle (Co), LPS, Dex or LPS+Dex in the presence or absence of 10 μM SB203580 (p38 MAPK inhibitor). (**c**) BMDMs from WT and *p38α*^*Mx1Cre*^ mice were treated for 2 h with vehicle (Co), LPS, Dex or LPS+Dex, then SphK1 mRNA expression was analysed. (**d**) Quantitative analysis of SphK1 mRNA expression in BMDMs treated for 2 h with vehicle (Co), LPS, Dex or LPS+Dex in the presence or absence of 10 μM SB747651A (MSK1 inhibitor). (**e**) The direct (physical) and indirect (functional) association among the downregulated genes after MSK1 inhibition is achieved using the STRING 10.1 database, and finally the network is visualized using Cytoscape 3.1.0. Results are presented as mean±s.e.m. Number of technical replicates: (**a**) (3); number of biological replicates: (**b**) (3), (**c**) (3 or 4) and (**d**) (6–8). Statistical analysis was performed by one-way analysis of variance. **P<0.01 and ***P<0.001; NS, not significant.

As the induction of SphK1 expression requires an intact GR dimerization interface, we examined the physical presence of the GR on upstream promoter elements of the *SphK1* gene. Using chromatin immunoprecipitation (ChIP), we demonstrated direct binding of the wild-type GR to a putative GR-binding site of the *SphK1* gene (Fig. 5a and Supplementary Fig. 6a). In contrast, the *GR^dim^* receptor was not recruited to this binding site after treatment with Dex or LPS+Dex (Fig. 5a). Moreover, co-treatment with LPS and Dex did not further increase GR DNA binding in wild-type cells relative to Dex alone (Fig. 5a). This suggests that the synergistic induction of SphK1 mRNA expression by LPS and Dex is instead related to a regulative effect by LPS.

**Figure 6:**
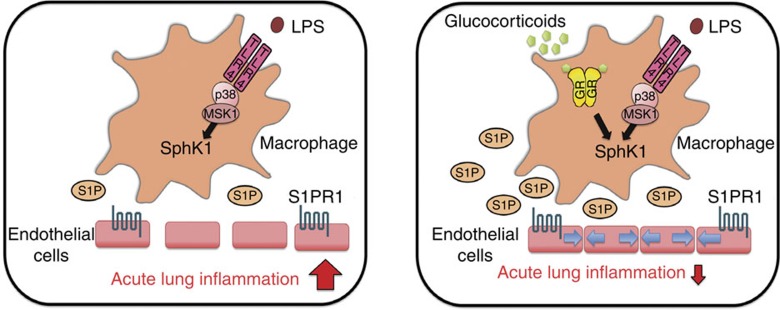
GCs and LPS regulate SphK1 and S1P dependent on GR dimerization and the p38–MSK1 pathway in macrophages to limit ALI. During inflammation, SphK1 is moderately expressed. This results in a low activation of S1PR1, an increase in lung endothelial barrier permeability and exacerbation of ALI (left). During GC treatment of ALI, SphK1 expression in macrophages is synergistically induced by the GR- and LPS-triggered signalling via activation of TLR4 p38 MAPK–MSK1. This leads to an elevation of S1P, which acts on S1PR1 to decrease lung endothelial barrier permeability. This enhanced S1P level is a prerequisite to limit ALI (right).

We therefore tested the involvement of signalling molecules downstream of the LPS/TLR4 pathway in the synergistic regulation of *SphK1*. LPS-induced IkB kinase 2 (IKK-2) signalling and subsequent NF-κB activation are not involved in SphK1 regulation because treatment with the IKK inhibitor BMS345541 had no effect, and the synergistic induction of SphK1 mRNA was not affected in BMDMs lacking RelA (Supplementary Fig. 6b,c). In contrast, we identified p38 MAPK as an essential component of the synergistic induction by LPS+Dex. Treatment of BMDMs with the p38 MAPK inhibitor SB203580 strongly interfered with the synergistic upregulation of SphK1 by LPS+Dex at 2 and 4 h (Fig. 5b and Supplementary Fig. 6d). Contrarily, the p38 MAPK inhibitor did not interfere with SphK1 mRNA induction by Dex alone (Fig. 5b and Supplementary Fig. 6d). Accordingly, synergistic induction of SphK1 by LPS+Dex was attenuated in BMDMs derived from mice with inducible knockout of p38 MAPK (*p38α^Mx1Cre^*; Fig. 5c). A similar finding was observed for the involvement of MSK1, a downstream target of p38 MAPK, in the regulation of SphK1 expression. Treatment of BMDMs with the MSK1 inhibitor SB747651A diminished the induction of SphK1 mRNA by LPS+Dex (Fig. 5d). To obtain additional insight into the mechanism of synergistic SphK1 induction by LPS+Dex, we used the Phospho Explorer Antibody Array as a means of identifying direct or indirect MSK1 downstream targets. BMDMs were treated with or without SB747651A, stimulated with LPS+Dex and subsequently analysed concerning the protein phosphorylation status. Using this method, we identified 16 top candidate proteins with decreased phosphorylation upon MSK1 inhibition in LPS+Dex-stimulated BMDMs, which might represent direct or indirect downstream targets (Table 1). Strikingly, a number of these targets interact with Src signalling, which may be a regulator of the cooperative LPS and Dex signalling (Fig. 5e). Taken together, the results show that LPS-induced p38 MAPK and MSK1 trigger a signalling network cooperate with the dimerized GR to synergistically induce *SphK1* gene expression (Fig. 6).

## Discussion

Treatment of ALI with GCs is highly debated because of conflicting data emerging from clinical studies and trials^4^,^35^. Delayed initiation of high-dose GC treatment may be detrimental because of the occurrence of muscle weakness and the possibility of severe secondary infections^3^,^8^. In contrast, GC treatment during the early exudative phase of ALI might be beneficial because it can resolve the acute phase of lung inflammation^6^,^7^,^8^. We present here, for the first time, the molecular mechanism of the anti-inflammatory effects of GCs during the early phase of ALI.

We have identified a novel mechanism by which GCs interfere with the pathogenesis of ALI, involving increased *SphK1* gene expression and S1P production. The SphK1–S1P–S1PR1 axis is recognized as an important regulator of endothelial barrier integrity that prevents lung inflammation by binding S1P to S1PR1 on lung endothelial cells^21^,^22^,^23^,^36^,^37^. We provide evidence that GR-dependent *SphK1* expression in myeloid cells, particularly macrophages, is responsible for the elevated levels of S1P in plasma and the subsequent suppression of ALI. First, neither *GR*^*LysMCre*^ nor *GR*^*dim*^ mice had increased circulating S1P plasma levels after GC treatment, which correlates with the inability of Dex to suppress vascular leakage in the lungs of these two mouse strains. The Dex response was also attenuated in *GR*^*CD11cCre*^ mice, in which GR expression in neutrophils is retained. Hence, the GR in the monocyte/macrophage cell lineage is required for its anti-inflammatory effects. Second, in animals with ALI, treatment with the S1PR1 agonist SEW2781 or with Dex prevented vascular leakage to similar degrees. Indeed, SEW2781 even rescued vascular leakage in *GR*^*LysMCre*^ mice, which are refractory to Dex. Third, pharmacological inhibition of SphK1 in wild-type mice by co-treatment with DMS completely abrogated the inhibition of vascular leakage and suppression of inflammation by Dex. Finally, ablation of the *SphK1* gene in the myeloid lineage abolished the elevation of plasma S1P levels by Dex and eliminated any effects of GCs on vascular leakage and inflammation. In addition to macrophages, other sources of S1P production are known, such as red blood cells thrombocytes and endothelial cells^38^. Hence, it is likely that these cells also contribute to the basal plasma levels of S1P. Accordingly, we detected induction of SphK1 in endothelial cells by Dex; however, synergistic regulation by LPS and Dex was not observed. Although we cannot entirely exclude a contribution from endothelial cells to Dex-regulated S1P plasma levels, the lack of S1P induction in Dex-exposed myeloid-specific GR and SphK1 mutant mice strongly indicates that macrophages make a major contribution to this GC effect.

Our data support the concept that SphK1 expression and hence S1P production are induced by GCs under inflammatory conditions and are required for the anti-inflammatory actions of GCs. Despite earlier reports suggesting that SphK1 has a pro-inflammatory role^39^,^40^ recent evidence affirms a major anti-inflammatory function of SphK1 (ref. 41). Specifically, mice with complete deletion of *SphK1* (*SphK1*^*−/−*^) are highly susceptible to LPS-induced ALI and exhibit increased lung vascular leakage. This suggests that the SphK1 function is essential for protection from lung injury. Accordingly, viral SphK1 overexpression protects these mice from LPS-induced ALI^41^. Moreover, S1P has recently been shown to mediate anti-inflammatory effects in chronic inflammatory diseases as well, such as psoriasis^42^. In addition, patients with multiple sclerosis have been efficiently treated with FTY720, an S1P analogue^43^,^44^. An ongoing clinical trial is investigating the effects of FTY720 on lung function (NCT00416845), which highlights the importance of our attempt to identify the molecular mechanism that stabilizes the SphK1–S1P–S1PR1 axis. We demonstrate that SphK1 expression in macrophages is sufficient to protect against ALI without having any effect on the composition of leukocyte subpopulations, plasma cytokines and BMDM cytokine mRNA expression. This is in agreement with the findings of Xiong *et al*.^45^. Strikingly, we observed that Dex efficiently represses cytokine secretion in *SphK1*^*LysMCre*^ mice, indicating that cytokine repression is not sufficient to resolve inflammation. Our results prove that the SphK1–S1P axis plays an essential role in attenuating lung inflammation by GCs. Moreover, we provide evidence that reducing the expression of pro-inflammatory cytokines, a classic feature of Dex treatment, is not sufficient to resolve ALI. Finally, we show that the abundance of individual leukocyte subsets in the blood was largely similar in wild-type and *GR*^*dim*^ mice, albeit the latter ones are refractory to Dex treatment. The sole difference we observed was that Dex increased the number of neutrophil granulocytes only in the blood of wild-type and not of *GR*^*dim*^ mice. Granulocytosis after GC therapy is a well-known phenomenon and has been assigned to the demargination of these cells from the blood vessels endothelium^46^. As we could show that direct effects of GCs on neutrophils were not involved in the suppression of ALI, it is possible that changes in endothelial cells induced by increased S1P plasma levels may be responsible for the granulocytosis observed in wild-type mice only. Collectively, these findings indicate that modulation of the immune system by GCs does not contribute to the resolution of lung inflammation.

It has been reported that LPS^47^,^48^ and Dex alone^49^,^50^can induce SphK1 mRNA expression. However, combined treatment with LPS and Dex—which mimics exposure to GCs during inflammatory conditions—led to a strong synergistic upregulation of SphK1 mRNA expression. This strong induction was seen in primary BMDMs and was dependent on an intact GR dimerization interface. Hence, the wild-type GR but not the mutant *GR*^*dim*^ receptor is recruited to the *SphK1* gene after Dex and LPS+Dex treatment; however, as recently shown, the *GR*^*dim*^ can still bind preferentially as a monomer to some sites compromising the GR cistromes^51^. Interestingly, the synergistic regulation appears to be restricted to macrophages, as we did not observe a similar effect in endothelial cells, where Dex-stimulated SphK1 expression independently of LPS. Furthermore, we demonstrate for the first time that the synergistic action by pro-inflammatory stimuli and Dex on SphK1 mRNA upregulation occurs through a number of TLR ligands and is not restricted to TLR4 stimulation by LPS. Namely, TNFα, TLR1/2 and TLR3 agonists potently increased SphK1 mRNA levels in combination with Dex exposure in a manner similar to that of LPS. Moreover, our report of synergistic gene regulation being an anti-inflammatory mechanism is in line with the recent finding that the MAPK phosphatase DUSP1 is cooperatively induced by IL-1β and Dex in human pulmonary epithelial A549 cells^52^.

Our efforts to dissect the signalling pathways involved in the synergy between LPS and GCs revealed that NF-κB signalling was dispensable, whereas the p38 MAPK–MSK1 signalling axis was essential. In view of the established pro-inflammatory role of p38 (ref. 53), it might be considered counter-intuitive that synergy is required between p38 and the anti-inflammatory actions of GCs to mediate the upregulation of SphK1. In certain inflammatory models, p38 has been assumed to be a direct target of GC-induced DUSP1, resulting in the dampening of inflammation^17^. However, more recently, an anti-inflammatory function of p38 has also been demonstrated in some types of inflammation. This is illustrated by the worsened arthritis in mice with conditional deletion of p38 in myeloid cells^54^. Strikingly, in a mouse lung injury model, a p38 inhibitor failed to attenuate inflammation^55^. This is in line with our data that support the concept that p38 activation may contribute to the anti-inflammatory actions of GCs in ALI. An obvious candidate p38 target leading to SphK1 induction is MSK1, a kinase that phosphorylates many transcription factors and chromatin components^56^. The direct substrate of MSK1 involved in SphK1 regulation is unknown. Potential MSK1 downstream targets that might mediate the synergistic induction of SphK1 by LPS+Dex were identified by using the Phospho Explorer Antibody Array. Notably, one of the 16 top proteins showing decreased phosphorylation upon MSK1 inhibition and LPS+Dex stimulation is Src. We consider this protein to be a highly promising candidate as it has been shown to mediate HuR phosphorylation and hence stabilize SphK1 mRNA^57^, and is at the centre of the identified signalling network (Fig. 5e). Further investigation is required to fully understand SphK1 induction, for instance, to determine whether particular transcription factors are involved, for example, the GR itself^58^,^59^, or whether decondensation of chromatin occurs at the promoter of the *SphK1* gene to enhance accessibility for transcription factors.

Our results support the use of corticosteroids to activate the GR-coordinated SphK1–S1P–S1PR1 axis as an efficient treatment regimen in the early states of ALI. Furthermore, strengthening the SphK1–S1P–S1PR1 axis in combination with GC application might become a novel strategy for ALI therapy, and thereby enhance the efficacy of short-range GC treatment in septic patients since 40% of them develop ALI. Indeed, in recent studies, S1PRs were considered as potential biomarkers for the grade of lung inflammation^60^,^61^. Finally, the S1P analogue FTY720 is already in use for the treatment of multiple sclerosis^43^,^44^, and there are currently clinical trials studying the effects of FTY720 on lung function (NCT00416845). Furthermore, the use of Dex to treat lung inflammation is already at the clinical trial stage (NCT01731795), highlighting the importance of our findings on *SphK1* as a new GC-induced gene. Taken together, our findings suggest that targeting lung vascular stability mediated by p38–MSK1 signalling and the SphK1–S1P–S1PR1 axis might represent a new strategy for the treatment of lung inflammation.

In conclusion, our study provides novel insights into GC action that may pave the way for improved anti-inflammatory therapy of ALI.

## Methods

### Mice

*GR*^*dim*^ mice^62^ were backcrossed for at least five generations to the FVB/N background. *GR*^*LysMCre*^ mice^16^ were backcrossed for at least five generations to the BALB/c or C57BL/6 background. S*phK1*^*LysMCre*^ C57BL/6 mice were bred by Thorsten Schinke (Universitätsklinikum Hamburg-Eppendorf (UKE), Hamburg, Germany) and the *SphK1^flox^* mice were generated and kindly provided by Shaun R. Coughlin (University of California San Fransisco (UCSF), CA, USA). Mx1-cre *p38α^flox^* mice have previously been described^63^,^64^. To induce deletion of the floxed alleles in *Mx1-cre p38α*^*flox*^ mice, they were injected intraperitoneally (i.p.) with polyIC (13 mg kg^−1^ body weight, three times every 48 h). The mice were kept under pathogen-free conditions. Male and female mice at the age of 9–14 weeks were used.

Nomenclature for the used mouse strains:

*GR^flox^ (Nr3c1^tm2Gsc^), GR^LysMCre^ (Nr3c1^tm2Gsc^Lyz2tm1(cre)lfo/J)*,

*GR^CD11cCre^ (Nr3c1^tm2Gsc^Tg(Itgax-cre)1-1Reiz), GR^dim^ (Nr3c1^tm3Gsc^), SphK1^flox^ (SphK1^tm2Cgh^), SphK1^LysMCre^ (SphK1^tm2Cgh^Lyz2tm1(cre)lfo/J).*

### ALI and analysis

LPS was applied i.p. and OA was applied intravenously (i.v.). Male and female mice at the age of 9–14 weeks were injected i.p. with 10 mg kg^−1^ body weight LPS (Sigma, L2880), and 30 min later i.v. with 2.6 μl g^−1^ body weight OA (Sigma, O1008). OA was prepared as a 4% solution in 0.1% BSA^65^. Dex (1.25 mg kg^−1^ body weight; Sigma, D2915) was applied intranasally simultaneously with i.p. injection of LPS. For SphK1 inhibition, mice were injected i.v. with 200 μM DMS (Biomol) as previously described^66^. S1PR1 agonist SEW2781 (Cayman) was injected i.v. (0.3 mg kg^−1^ body weight) as previously described^67^. Mice were killed after 18–24 h, and lungs were prepared for BAL, EB extraction or histology.

Immediately after killing the mice, BAL was collected by tracheal cannulation using 1 ml of cold PBS and then centrifuged (300*g*, 15 min, 4 °C). Following resuspension of the pellet and lysis of erythrocytes, total cell numbers were determined by automatic counting (Casy counter, Schärfe).

Pulmonary microvascular permeability was determined by accumulation of EB dye (Sigma, E2129). EB dye (25 mg kg^−1^ body weight) was injected i.v. 30 min before killing. Following killing, the mice were perfused by right ventricle puncture with ice-cold PBS+5 mM EDTA. Lungs were removed and photographed, and EB dye was extracted in formamide (4 ml g^−1^) at 60 °C overnight. The supernatant was separated by centrifugation at 5,000*g* for 30 min at room temperature, and absorbance was measured by spectrophotometry at a wavelength of 620 nm (Mithras LB940, Berthold Technologies).

For histological analysis, lungs were perfused with PBS+5 mM EDTA and fixed in 4% formaldehyde in 0.1 M phosphate buffer (pH 7.2). Within 24 h, lungs were embedded in paraffin and 4-μm-thick sections were stained with haematoxylin/eosin. The histopathology of the lung injury was scored quantitatively as previously described^32^. The score is based on the following features: structure of the alveolar septae (thin or congested); intra-alveolar haemorrhage or fibrin; cell infiltrates (in septae, alveolar lumen, pleura and bronchi)^32^. Lung injury was characterized by patchy areas. Histological scoring was performed blindly. Inflammation was graded from 0 to 3, and other parameters of inflammation (vessel, pleura and bronchus) were evaluated. The average score was calculated as previously described in ref. 32.

### Extraction and quantification of S1P

S1P of mouse plasma was determined by liquid chromatography coupled to triple-quadrupole mass spectrometry (LC/MS/MS)^68^. Positive electrospray ionization LC/MS/MS analysis was used for detection of all analytes. Multiple reaction monitoring transitions were as follows: S1P *m/z* 380/264, C17-S1P *m/z* 366/250, sphingosine *m/z* 300/282, C17-sphingosine *m/z* 286/268 and sphingosine-D7 *m/z* 307/289. Liquid chromatographic resolution of all analytes was achieved using a 2 × 60 mm MultoHigh C18 reversed-phase column with 3 μm particle size (CS-Chromatographie Service). Standard curves were generated by adding increasing concentrations of the analytes to 300 pmol of the internal standards C17-sphingosine and C17-S1P or deuterated sphingosine-D7. Linearity of the standard curves and correlation coefficients were obtained by linear regression analyses. Data analysis was performed using Analyst 1.4 (AB Sciex).

### Cell culture and quantitative RT–PCR

BMDMs were obtained from wild-type and GR^dim^ mice as previously described^16^. BMDMs were treated with LPS (100 ng ml^−1^) and Dex (10^−6^ M) for the indicated durations. RNA isolation and quantitative RT–PCR analysis were performed as previously described^16^. The primers used for RT–PCR were SphK1 5′- CCAAGTGCACCCAAACTACC -3′ and SphK1 3′- GCCCCACCTTCTAGCTTTCT -5′. For inhibition experiments, BMDMs were pretreated for 30 min with 10 μM SB203580 (Sigma, S8307), a p38 MAPK pathway inhibitor; 10 min pretreated with 8 μM BMS345541 (Calbiochem), an IKKβ inhibitor; or 60 min pretreated with 10 μM SB747651A (Axon-Medchem), an MSK1 inhibitor. The cells were subsequently stimulated with LPS, Dex or LPS+Dex for the indicated durations. TNFα (500 units per ml; a gift from Claude Libert, VIB/University of Ghent, Belgium), Pam3CSK4 (200 ng ml^−1^; Invivogen), or polyIC (50 ng ml^−1^) was used either in a single treatment or together with Dex (10^−6^ M) to stimulate BMDMs for the indicated durations. RNA isolation and quantitative RT–PCR analysis were performed as previously described^16^. SphK1 activity in BMDMs was measured from the conversion of C17- and C18-sphingosine to S1P by LC/MS/MS as outlined above (Extraction and quantification of S1P) and as previously described^69^. The endothelial cell line TC10 was a gift from Claude Libert, VIB/University of Ghent, Belgium. Primary lung endothelial cells were obtained from Cell Biologics.

### Chromatin immunoprecipitation

ChIP analysis of wild-type and *GR*^*dim*^ BMDMs was performed 4 h after stimulation with LPS (100 ng ml^−1^) and Dex (10^−6^ M). ChIP assays were performed as described elsewhere^70^. The anti-GR antibody (sc-8992) was obtained from Santa Cruz Biotechnology. The following primers were used: SphK1 forward 5′- GCTGCTGATGTGAAGGATAC -3′ and SphK1 reverse 5′- GCTGCTGATGTGAAGGATAC -3′. Foxl2 was selected as a negative control and the following primers were used: forward 5′- GCTGGCAGAATAGCATCCG -3′ and reverse 5′- TGATGAAGCACTCGTTGAGGC -5′. The fold enrichment over immunoglobulin G was calculated. Putative GC-response element-binding sites were identified by Genomatix software.

### Flow cytometric analysis and sorting

Blood samples were obtained by cardiac puncture. A 100-μl aliquot of blood was collected into 900 μl Alsever's solution and then centrifuged at 350*g* for 10 min at 4 °C. The supernatant was removed, the cells were treated twice with erythrocyte lysis buffer, washed with 0.1% BSA in PBS and then resuspended in the reflux. Cell counts were determined with the aid of a Neubauer haemocytometer.

For flow cytometric analysis, Fc receptor blockade was performed by incubation with TruStain fcX (anti-mouse CD16/32; clone: 93; cat. no. 101320; 1:2,000). Subsequently, the cells were stained with the following monoclonal antibodies: PerCP/Cy5.5 anti-mouse CD3 (clone: 17A2; cat. no. 100218; 1:500), APC anti-mouse/human CD45R/B220 (clone: RA3-6B2; cat. no. 103212; 1:800), PE anti-mouse CD49b (clone: DX5; cat. no. 108908; 1:400), FITC anti-mouse F4/80 (clone: BM8; cat. no. 123108; 1:500), APC/Cy7 anti-mouse Ly-6G/Ly-6C (Gr-1; clone: RB6-8C5; cat. no. 108424; 1:2000), PE/Cy7 anti-mouse CD11c (cat. no. 117318; 1:500), PE anti-mouse CD115 (cat. no. 135505; 1:500). All the antibodies were obtained from BioLegend (Uithoorn, The Netherlands). Data were acquired on a BD FACS Canto II flow cytometer (BD Biosciences) and were analysed using FlowJo software (Tree Star).

### Protein phosphorylation profiling

Hybridization and analysis of the Phospho Explorer Antibody Array were carried out by Full Moon BioSystems (Sunnyvale, CA, USA) and served to survey the phosphorylation state of select proteins. The assay was performed using BMDMs pretreated for 60 min with 10 μM SB747651A, followed by LPS+Dex for 4 h. At the end of the incubation time, proteins were collected and analysed. Physical and functional association among the downregulated genes after MSK1 inhibition was done by using the STRING 10.1 database and the network was visualized using Cytoscape 3.1.0.

### Statistics

Results are presented as mean±s.e.m. Statistical analyses were performed with one-way analysis of variance (ANOVA) for comparison, followed by Tukey's *post hoc* within one group. For the comparison of two groups (genotypes), one-way analysis of variance followed by Tukey's *post hoc* test for multiple comparisons was performed. Statistical analyses were performed using the GraphPad Prism software (GraphPad Software) and sample size was chosen with G*Power3.1 (University Düsseldorf). Outlying sample exclusion criteria were done with GraphPad Prism Outlier Calculator. Data were considered statistically significant: *P<0.05, **P<0.01 and ***P<0.001.

### Declaration of approval for animal experiments

All experiments involving animals were approved by the Thüringer Landesamt für Lebensmittelsicherheit und Verbraucherschutz (TLLV) and Regierungspräsidium Tübingen.

## Additional information

**How to cite this article:** Vettorazzi, S. *et al*. Glucocorticoids limit acute lung inflammation in concert with inflammatory stimuli by induction of SphK1. *Nat. Commun.* 6:7796 doi: 10.1038/ncomms8796 (2015).

## Supplementary Material

Supplementary Information Supplementary Figures 1-6

## Figures and Tables

**Table 1:** Downregulation of phosphorylation of distinct proteins after LPS+Dex treatment and MSK1 inhibition determined by phospho-array.

**Gene name**	**Target**	**Phospho-expression**	**Fold change**
*Ccnd3*	Cyclin D3 (p-Thr283)	Down	0.694
*Crk*	CrkII (p-Tyr221)	Down	0.681
*Hdac1*	HDA C1 (p-Ser421)	Down	0.545
*Hsp90ab1*	HSP90B (p-Ser226)	Down	0.664
*Hspb2*	HSP27 (p-Ser82)	Down	0.723
*Kdr*	VEGFR2 (p-Tyr951)	Down	0.613
*Lyn*	LYN (p-Tyr507)	Down	0.674
*Nf2*	Merlin (p-Ser10)	Down	0.557
*Raf1*	Raf1 (p-Ser259; p-Ser289)	Down	0.404
*Rps6ka1*	P90RSK (p-Thr359/Ser363; p-Thr573)	Down	0.626
*Rps6ka5*	MSK1 (p-Ser360)	Down	0.730
*Src*	Src (p-Tyr529)	Down	0.641
*Syk*	SYK (p-Tyr525)	Down	0.697
*Ywhaz*	14-3-3 zeta (p-Ser58)	Down	0.720
*Pgr*	Progesterone receptor (A b-190)	Down	0.756
*Krt18*	Keratin 18 (A b-52)	Down	0.779
*Jun*	c-Jun (A b-93)	Down	0.781

BMDMs, bone marrow-derived macrophages; Dex, dexamethasone; LPS, lipopolysaccharide.

BMDMs were pretreated for 60 min with and without 10 μM SB747651A, and then treated with LPS+Dex for 4 h, and changes in protein phosphorylation events were measured with the Phospho Explorer Antibody Array (Full Moon BioSystems). The table lists proteins where the expression of protein phosphorylation was downregulated after MSK1 inhibition. The fold change indicates the change of downregulation.
