# Supplementary material for: Glucocorticoids limit acute lung inflammation in concert with inflammatory stimuli by induction of SphK1
Source: Nat Commun. 2015 Jul 17;6:7796. doi: 10.1038/ncomms8796 (PMC4518295; doi:10.1038/ncomms8796)
Supplement: Supplementary Information — Supplementary Figures 1-6 [file ncomms8796-s1.pdf]

Supplementary Information

Supplementary Figure 1

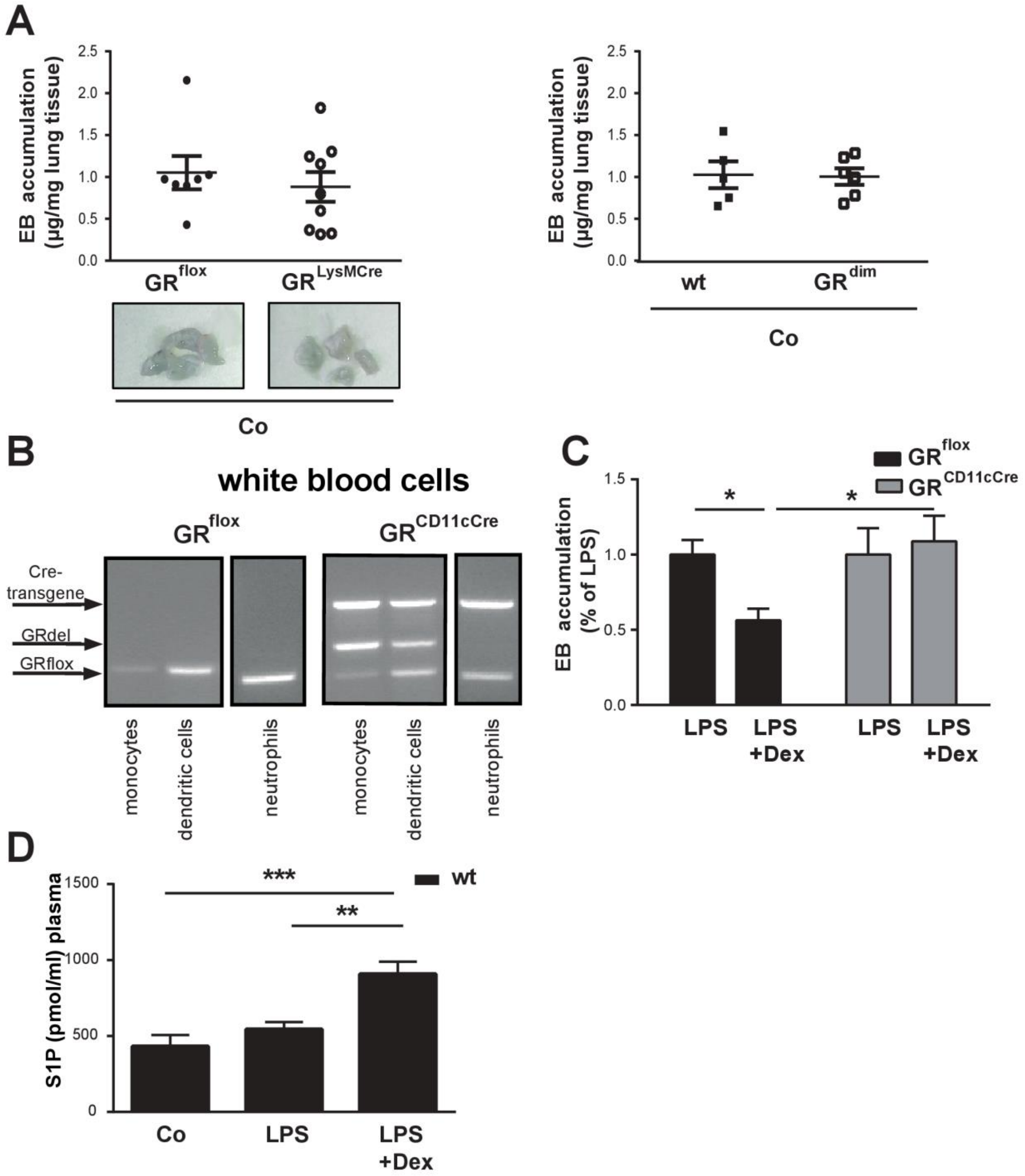

Supplementary Figure 1.
EB accumulation in GR^{LysMCre}, GR^{dim} and GR^{CD11cCre} mice, recombination of white blood cells in GR^{CD11cCre} mice and S1P plasma levels in wt mice.
(**A**) *GR*^{flox} and *GR*^{LysMCre} mice and wild type (wt) and *GR*^{dim} mice were treated with vehicle (Co) for 18 h/24 h. Evans Blue dye (EB) was injected i.v. 30 min before sacrifice, then EB accumulation was quantified after perfusion (PBS + 5 mM EDTA). (**B**) From *GR*^{flox} and *GR*^{CD11cCre} mice white blood cells were sorted by flow cytometry for monocytes (CD115^{+}; F4/80^{+}; GR-1^{low}; CD11c^{+}), dendritic cells (CD115^{-}; F4/80^{-}; CD11c^{+}) and neutrophils (GR-1^{+}). PCR was subsequently performed on genomic DNA with primers amplifying the cre transgene (600 bp), the GRflox allele (275 bp) and the GR deleted allele (390 bp). (**C**) *GR*^{flox} and *GR*^{CD11cCre} mice were injected with LPS or LPS and Dex (LPS+Dex) for 18 h. EB was injected i.v. 30 min before sacrifice and quantified as described in Fig. 1A. (**D**) Wt mice were injected with vehicle (Co), LPS, or LPS+Dex. After 24 h, the S1P level in plasma (pmol/ml) was determined by LC/MS/MS. Results are presented as mean ± SEM. Number of biological replicates: A (5–9), D (4–6), E (14–21). In (E) data are from six independent biological experiments. Statistical analysis was performed by one-way ANOVA. * $p < 0.05$; ** $p < 0.01$; *** $p < 0.001$.

Supplementary Figure 2

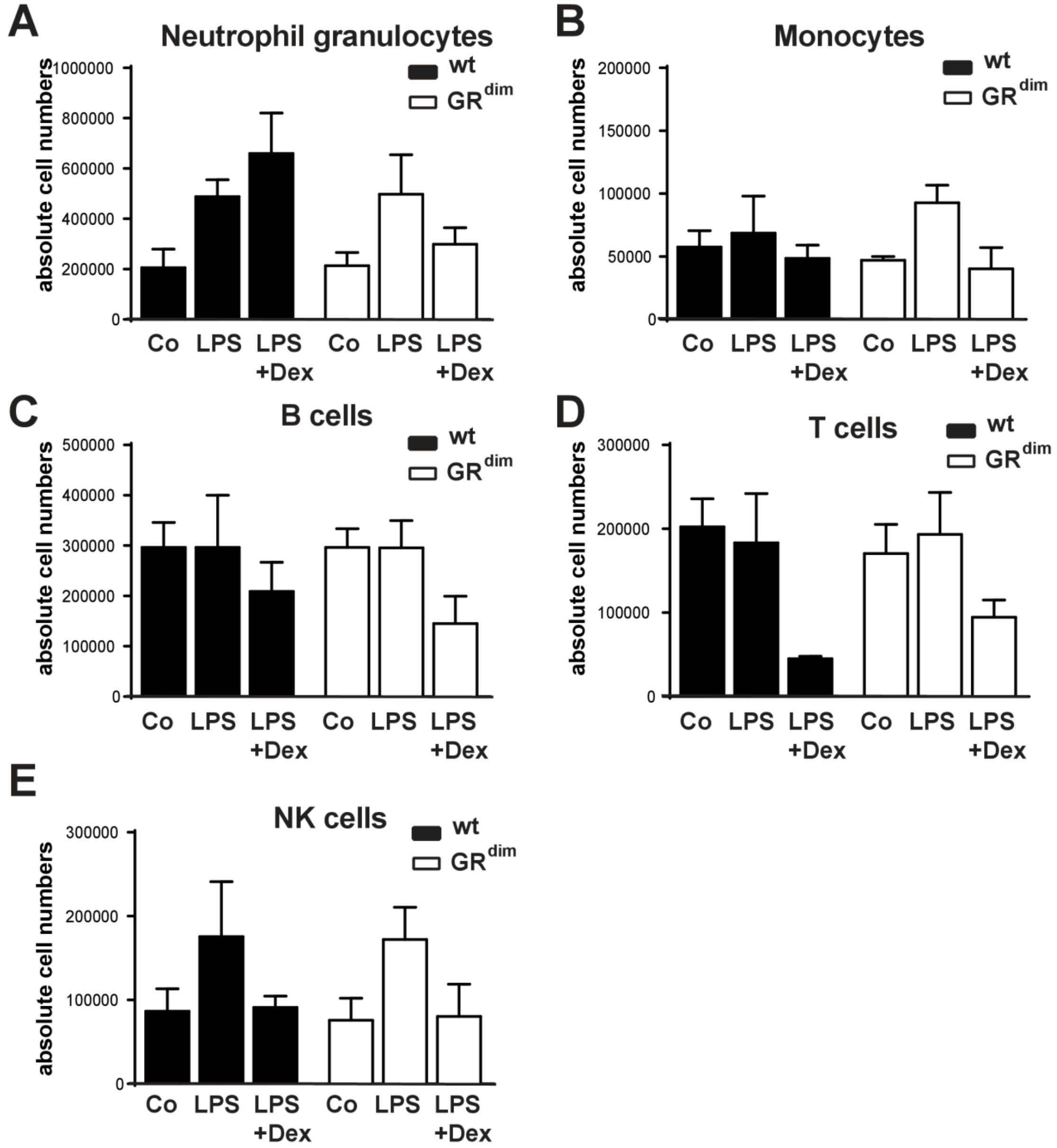

Supplementary Figure 2.
Leukocyte populations during ALI in wt and GR^{dim} mice
Wt and *GR^{dim}* mice were treated as indicated in Fig. 1A. The abundance of individual leukocyte subpopulations in the blood was determined 24 h after the indicated treatments by flow cytometry in combination with cell counting using a haemocytometer. Absolute cell numbers of (**A**) neutrophil granulocytes (GR-1^{high}), (**B**) monocytes (F4/80^{+}, GR-1^{low}), (**C**) B cells (CD45R/B220^{+}), (**D**) T cells (CD3^{+}) and (**E**) Natural killer (NK) cells (CD49b^{+}) in 100 µl blood are shown in the graphs. Data are depicted as mean ± SEM. Number of biological replicates: 4 or 5 per group.

Supplementary Figure 3

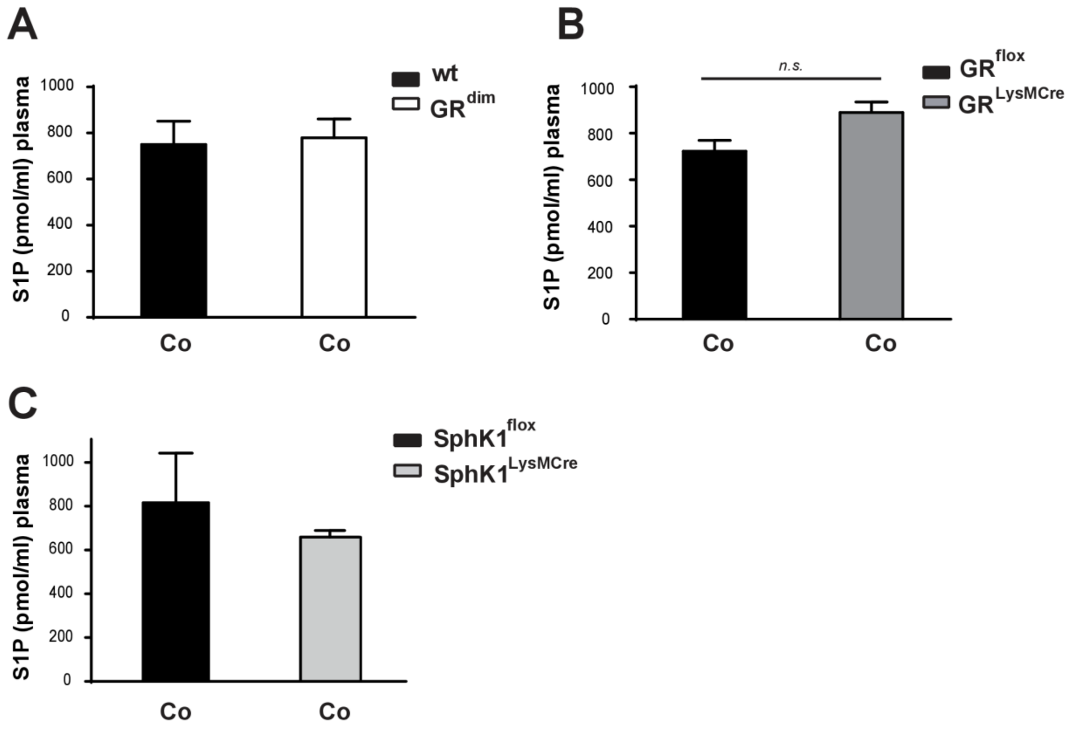

Supplementary Figure 3.

Basal S1P plasma levels in GR^{dim}, GR^{LysMCre}, SphK1^{LysMCre} and the littermate control animals

(**A**) The S1P level in plasma (pmol/ml) was determined in *GR^{dim}* and wt littermate control mice by LC/MS/MS 24 h after treatment with vehicle. (**B**) The S1P level in plasma (pmol/ml) was determined in *GR^{flox}* and *GR^{LysMCre}* mice by LC/MS/MS 18 h after treatment with vehicle. (**C**) The S1P level in plasma (pmol/ml) was determined in *SphK1^{flox}* and *SphK1^{LysMCre}* mice by LC/MS/MS 24 h after treatment with vehicle. Results are presented as mean ± SEM. Number of biological replicates: A (11–13), B (5 or 6), C (6 or 7). Statistical analysis was performed by one-way ANOVA. n.s.: not significant.

Supplementary Figure 4

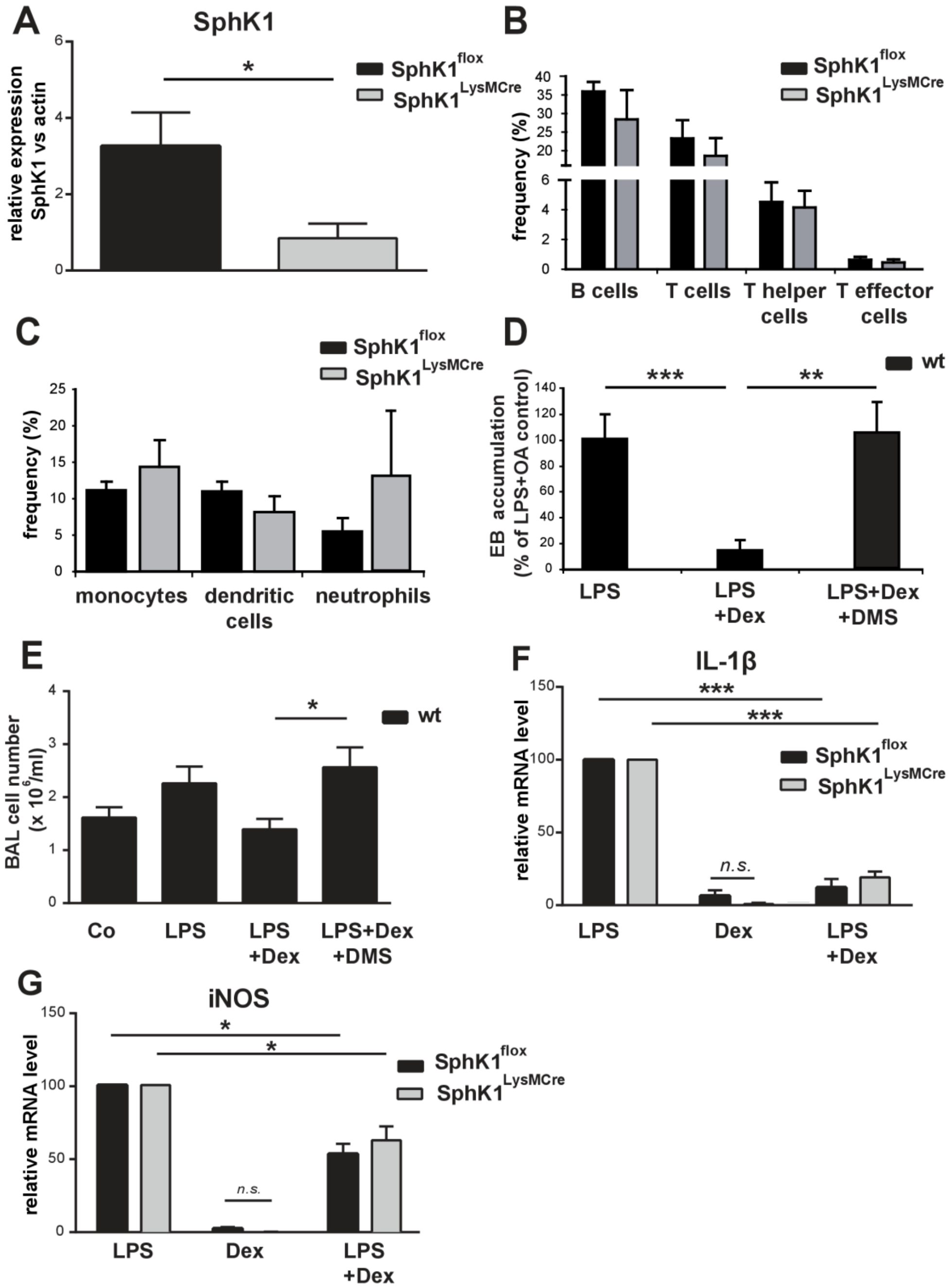

Supplementary Figure 4.

Characterization of SphK1^{LysMCre} mice and inhibition of SphK1 in vivo

(**A**) BMDMs derived from *SphK1^{flox}* and *SphK1^{LysMCre}* mice were analysed for SphK1 mRNA expression by quantitative RT-PCR. (**B, C**) White blood cells from naive *SphK1^{flox}* and *SphK1^{LysMCre}* mice were analysed by flow cytometry (gated for lymphocytes and single cell frequency) for (**B**) lymphocytes: B cells (B220^{+}), T cells (CD3^{+}), T helper cells (CD3^{+}; CD4^{+}), T effector cells (CD3^{+}; CD8^{+}), and (**C**) myeloid cells: monocytes (CD115^{+}; CD11b^{+}), myeloid dendritic cells (CD11c^{+}; CD11b^{+}; CD115^{+}), neutrophils (SSC^{high}; GR-1^{high}; CD11b^{+}). (**D, E**) Wt mice were injected with LPS or LPS+Dex. An additional LPS+Dex-treated group received DMS (an SphK1 inhibitor) for a 24-h time period. (**D**) EB was injected 30 min before sacrifice and EB accumulation was quantified after perfusion (PBS + 5 mM EDTA). (**E**) Cell numbers in the bronchoalveolar lavage (BAL) obtained from mice 24 h after treatment with vehicle (Co), LPS, LPS+Dex, or LPS+Dex+DMS. (**F, G**) BMDMs derived from *SphK1^{flox}* and *SphK1^{LysMCre}* mice were treated for 4 h with LPS, Dex, or LPS+Dex and (**F**) IL-1β mRNA and (**G**) iNOS mRNA were analysed by quantitative RT-PCR. Values were normalized to those of LPS-treated cells. Results are presented as mean ± SEM. Number of biological replicates: A (10 or 11), B (3), C (3), D (8–12), E (6 or 7), F and G (4 or 5). In (D) and (E) data are from two independent biological experiments. Statistical analysis was performed by one-way ANOVA. * $p < 0.05$; ** $p < 0.01$; *** $p < 0.001$; n.s.: not significant.

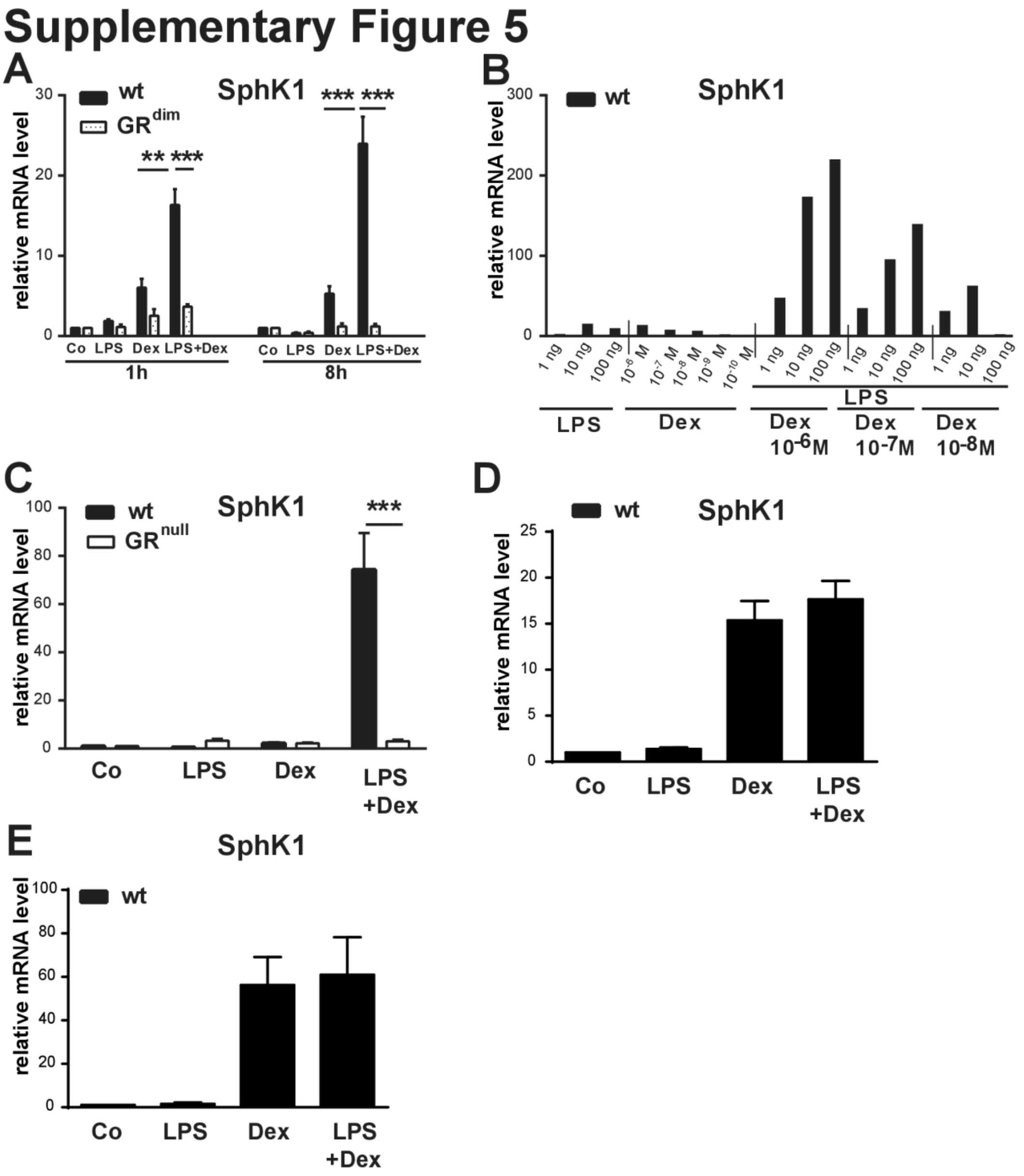

Supplementary Figure 5.

SphK1 expression in GR^{dim} – and GR^{null} -macrophages and in endothelial cells

(**A**) BMDMs derived from wt and GR^{dim} mice were treated with vehicle (Co), LPS, Dex, or LPS+Dex for the indicated durations and SphK1 mRNA was analysed by quantitative RT-PCR. (**B**) BMDMs derived from wt mice were treated for 4 h with the indicated concentrations of LPS, Dex, or LPS+Dex, and mRNA levels of SphK1 were analysed by quantitative RT-PCR. (**C**) Macrophages derived from fetal liver of wt and GR complete knockout (GR^{null}) mice were treated with vehicle (Co), LPS, Dex, or LPS+Dex for 4 h and mRNA levels of SphK1 were analysed by quantitative RT-PCR. (**D**) Endothelial cell line TC10 was treated with vehicle (Co), LPS, Dex, or LPS+Dex for 4 h and SphK1 mRNA expression was analysed by quantitative RT-PCR. (**E**) Primary mouse lung endothelial cells were treated with vehicle (Co), LPS, Dex, or LPS+Dex for 4 h and SphK1 mRNA expression was analysed by quantitative RT-PCR. Results are presented as mean ± SEM. Number of biological replicates: A (3–5), B (2 or 3), C (2 or 3), D (5), E (3). Statistical analysis was performed by one-way ANOVA. ** $p < 0.01$; *** $p < 0.001$.

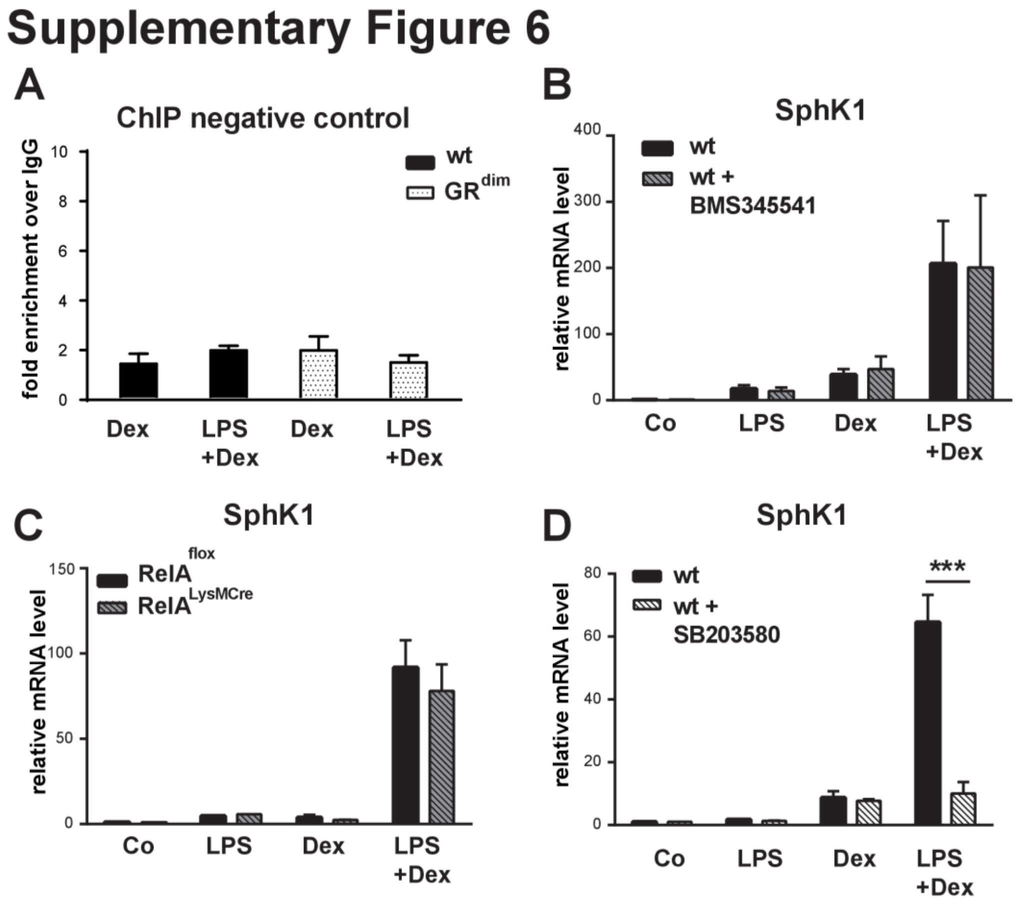

Supplementary Information Figure 6.
ChIP from macrophages and SphK1 expression after p38, MSK1 inhibition and in RelA knock out macrophages

(**A**) Chromatin immunoprecipitation of wt and *GR*^{dim} BMDMs was conducted 4 h after the indicated treatment (Dex, LPS+Dex). Shown are the quantitative RT-PCR results of the negative control (Foxl2). (**B**) BMDMs were treated for 2 h with vehicle (Co), LPS, Dex, or LPS+Dex in the presence or absence of 8 µM BMS344551 (MSK1 inhibitor) and mRNA levels of SphK1 were analysed by quantitative RT-PCR. (**C**) BMDMs from *RelA*^{flox} and *RelA*^{LysMCre} mice were treated for 2 h with vehicle (Co), LPS, Dex, or LPS+Dex, and mRNA levels of SphK1 were measured as in (B). (**D**) BMDMs were treated for 4 h with vehicle (Co), LPS, Dex, or LPS+Dex in the presence or absence of 10 µM SB203580 (p38 MAPK inhibitor) and SphK1 mRNA expression was analysed by quantitative RT-PCR. Results are presented as mean ± SEM. Number of technical replicates: A (3). Number of biological replicates: B (4), C (2 or 3), D (3). Statistical analysis was performed by one-way ANOVA. *** $p < 0.001$.
